# Transfusion and Autotransfusion

**Published:** 1877-02-20

**Authors:** 


### TRANSFUSION AND AUTO-TRANSFUSION.

Abstract of a lecture delivered by Dr. Lesser before the Berlin Gynæcological Society, December 1, 1874, and published in *Klinische Vortraege*, No. 86.

Three medical questions have excited general interest in Germany during the past few years, (1) military surgery (2), avoiding the loss of blood in operations, and (3) transfusion, which has been very generally discussed, though with little result. Here physiological experiment proves more profitable than clinical observation, and the author turns to that.

He begins with the account of a simple experiment. If the vena jugularis of a small dog be connected by a system of tubes and canulæ with the carotid of a large dog, the blood of the large animal passes over into the smaller, which soon begins to struggle, but then becomes quiet, and the activity of the respiration is diminished. The large dog at first remains still, but the loss of blood causes after a while a quivering of the muscles, the breath is drawn deeper and more rapidly. Somewhat later the cramps accompanying loss of blood begin. Meanwhile, in the small dog the vessels are found swollen, and the eyes projected. If the experiment be now interrupted, the smaller animal runs about, all right. The larger animal lies motionless, the flow of blood from the carotid has almost ceased. If the artery is closed, the head lowered and the limbs compressed, so as to drive the blood from the extremities and the abdominal cavity to the central regions, the animal begins to breathe again, and if the carotid is then re-opened the flow of blood begins anew, continuing till death follows. Upon weighing the small dog it was found, after the experiment described, that its quantity of blood had been doubled without producing any immediate harm.

This large addition of blood does not produce extravasations, but remains for the greater part in the vessels, as shown by Worm-Mueller. The arterial pressure is not, however, thereby increased, because the elasticity of the walls of the vessels is changed in a peculiar way ; the capacity of the vascular system is thereby increased sufficiently to take up the extra blood without any rise of pressure. As the limits of this power of self accommodation lie beyond the quantity of blood which might come into consideration in making a therapeutical transfusion, the fear of producing a dangerous rise in the pressure in the vessels by transfusion is unfounded, except, at most, in cases of certain diseases of organs in which any rise of the blood pressure might be followed by dangerous effects. The author makes other extremely important applications of this new discovery.

From animals whose blood has been doubled by transfusion, only a part of the blood can be recovered, and the animals *die* by bleeding *before the quantity of blood has reached the normal level.* If, however, the extremities be wrapped in

Esmarch's bandages and all means used to drive the blood towards the heart, the circulation recommences, and the pressure in the carotids, which was very low, rises again. In this way the life of the animal may be saved.

This method has been called auto-transfusion by the French, and seems destined to become of the greatest value, and has already been used with success, though not many trials have been made of it. The author enumerates the following indications for its use:

(1.) Small loss of blood, before having recourse to transfusion, and before and after surgical operations.

(2.) In cases of anæmia, before and after operations by which a fresh loss of blood is unavoidable.

(3.) Operations requiring the inhalation of chloroform, in cases of anæmia, as the pressure of the blood is lowered by the influence of the chloroform, Lentz, Brunner, Scheinesson, etc.

(4.) It should always precede transfusion itself, especially in cases of loss of blood, as by it life may be maintained during the critical moment, which is often lost in preparing the instruments for transfusion.

If the auto-transfusion suffices, it shows that a transfusion is unnecessary; and becomes in this way a good means of diagnosis.

The author then discusses the various forms of anæmia in their relations to the quantity of blood and its pressure.

In the author's experiments the transfusion was, of course, made with the natural blood. The principal danger in this case is that of coagulation or the introduction of air, which the author reduced to a minimum by using merely two canulæ, one for the artery and one for the vein, and connecting them by short bits of rubber tubing, with an intermediate glass tube. He recommends direct transfusion, and to avoid complicated apparatus. For indirect transfusion he considers a constant pressure of mercury, and that preliminary warming of the blood is unnecessary, as a cold temperature delays coagulation, and Malgaigne, Polli, and Casse found no harm to be done by the injection of blood at the ordinary temperature.

Since the introduction of defibrinated blood diminishes the coagulability, transfusion with it must be rejected when there is a fresh wound, or escape of blood.

Transfusion is a means of saving life, the loss of which is imminent either from certain acute diseases, want of blood, or asphyxia of the tissues. It is evident that for man undefibrinated human blood is the best, but the blood of animals may be used when it has no poisonous influence on the system. It is desirable to find some animal which may be obtained more readily than lambs, and the proposal to try dogs is worth experiment.

Dr. Lester ends his interesting and original lecture with a final recommendation of auto-transfusion.—*C. S. Minot: Boston Medical and Surg. Journal.*

